# HDL Composition, Heart Failure, and Its Comorbidities

**DOI:** 10.3389/fcvm.2022.846990

**Published:** 2022-03-08

**Authors:** Ahmed Diab, Carla Valenzuela Ripoll, Zhen Guo, Ali Javaheri

**Affiliations:** Division of Cardiology, Washington University School of Medicine, Saint Louis, MO, United States

**Keywords:** high-density lipoprotein (HDL), apolipoprotein A-I, apolipoprotein M, sphingosine-1-phosphate, heart failure, cardiomyopathy

## Abstract

Although research on high-density lipoprotein (HDL) has historically focused on atherosclerotic coronary disease, there exists untapped potential of HDL biology for the treatment of heart failure. Anti-oxidant, anti-inflammatory, and endothelial protective properties of HDL could impact heart failure pathogenesis. HDL-associated proteins such as apolipoprotein A-I and M may have significant therapeutic effects on the myocardium, in part by modulating signal transduction pathways and sphingosine-1-phosphate biology. Furthermore, because heart failure is a complex syndrome characterized by multiple comorbidities, there are complex interactions between heart failure, its comorbidities, and lipoprotein homeostatic mechanisms. In this review, we will discuss the effects of heart failure and associated comorbidities on HDL, explore potential cardioprotective properties of HDL, and review novel HDL therapeutic targets in heart failure.

## Introduction

Cardiovascular disease (CVD) is a leading cause of mortality worldwide (1). Heart failure (HF) is a common result of cardiometabolic disease and a major contributor to CVD mortality (2). The prevalence of HF in the developed world is rising and is estimated to be at 2%, while the incidence approaches 5–10 per 1,000 persons per year (3). HF is a clinical syndrome, typically presenting with symptoms of dyspnea, fluid retention, and decreased exercise tolerance. It usually follows structural or functional disorders of the endocardium, myocardium, or pericardium and is divided into three categories: HF with reduced ejection fraction (HFrEF), HF with preserved ejection fraction (HFpEF), and HF with mid-range ejection fraction (4, 5).

Multiple rationale suggest a mechanistic link between lipoproteins and HF. Interestingly, in HF patients, plasma cholesterol concentrations are inversely associated with mortality (6, 7). This observation, termed the “cholesterol paradox,” could be related to malnutrition, cachexia (8, 9), and inflammation (10–14) observed in HF patients, as well as direct effects of lipoproteins on the myocardium. Moreover, recent Mendelian randomization studies support a causal effect of low-density lipoprotein cholesterol (LDL-C) and triglycerides on LV mass and myocardial remodeling (15). Analogously, a clinical trial showed that reconstituted high-density lipoprotein (HDL) infusion shortens cardiac repolarization, demonstrating the capability of HDL to alter cardiac electrophysiological properties (16). Both studies exemplify a direct role of lipoproteins on the myocardium. Furthermore, lipoproteins can function as a fuel source, an important consideration in HF patients, where the energy-starved myocardium primarily consumes ketone bodies and fatty acids (17).

Based on two large randomized trials, a case could even be made for statin use in HF patients, thus LDL-C lowering *via* statins is unlikely to exacerbate HF outcomes (18, 19). We hypothesized that decreased HDL or HDL-associated apolipoproteins could be a driver of adverse HF outcomes (18, 19). High-density lipoprotein cholesterol (HDL-C) is inversely associated with CVD risk, as large epidemiological studies, such as the Framingham Heart Study have shown (20). Nonetheless, multiple randomized trials have failed to show a decrease in CVD risk or major adverse cardiac events when increasing HDL-C levels as a therapeutic target (21, 22). One interpretation of these findings is that, rather than the steady-state cholesterol mass, HDL or its associated apolipoproteins could exert beneficial effects in the setting of HF (or even CVD or other cardiac inflammatory disorders). For instance, our group has shown that reduced pre-transplant HDL cholesterol efflux capacity is associated with the progression of cardiac allograft vasculopathy, a major cause of mortality for cardiac transplant recipients (23). This example served as a proof-of-paradigm that HDL functions may be relevant outside of traditional atherosclerosis. The cardioprotective role of HDL may be related to its anti-oxidant and anti-inflammatory properties, endothelial protection, as well as its reverse cholesterol transport capacity (24).

Many pre-clinical studies performed mainly in rodents focus on the effect of HDL in cardiac pathophysiology and have shown positive effects on the myocardium. For instance, HDL can reduce infarct size in the setting of cardiac ischemia/reperfusion injury, attenuate apoptosis, preserve mitochondrial function, and protect the myocardium against oxidative stress (25–31).

Although a broad range of anti-atherogenic properties have been attributed to HDL, many are independent of its cholesterol content and reverse cholesterol transport. The heterogeneous properties of HDL particles are relatively complex, due to the wide variety of proteomic and lipidomic cargo of the particles. These characteristics lead to specific cardioprotective functions, such as increased endothelial nitric oxide (NO) production, reduced inflammation in endothelial cells and macrophages, stimulation of insulin-independent glucose uptake in the myocardium, among others. For example, the anti-oxidative capacity of HDL is mainly attributed to its ability to protect LDL from oxidation by free radicals. Of note, antioxidant components of HDL, such as the HDL-associated enzyme Paraoxonase 1 (PON1), metabolize lipid hydroperoxides and prevent their accumulation in LDL particles, decreasing LDL endocytosis by macrophages and formation of foam cells, thus averting the formation of atherosclerotic plaque (32–37).

Recent advances in proteomic characterization have led to the identification of novel HDL subclasses that will, in all likelihood, eventually supersede the historical size and density-based characterization system (38, 39). For historical reference, larger HDL2 particles are inversely associated with CVD risk, while smaller, denser HDL3 subclass exerts anti-atherogenic, anti-oxidant, and anti-inflammatory functions (40, 41), and these subclasses are also associated with mortality in acute HF patients. Total and small HDL particles (diameter <8.8 nm, mostly HDL3), measured by nuclear magnetic resonance spectroscopy, were inversely associated with 3-month mortality in patients with acute HF, while both large HDL and HDL-C demonstrated no significant association (42). Similarly, in HFrEF and HFpEF patients, total and small HDL were inversely associated with adverse outcomes (43).

To the best of our knowledge, the rigorous analysis of HDL proteomics has yet to be performed in advanced HF cohorts. Nonetheless, multiple preclinical and human epidemiological studies support the concept of pleiotropic effects of HDL-associated apolipoproteins (44, 45), which may play a significant role in the pathogenesis of HF. These observations led us to hypothesize that specific apolipoproteins and enzymes associated with HDL particles may potentially explain the cholesterol paradox and the underlying cardioprotective effects of HDL, which could be relevant therapeutic targets in HF. In this review, we will discuss the effects of HF and associated comorbidities on HDL, explore potential cardioprotective properties of HDL, and review novel HDL therapeutic targets in HF.

## Effects of Heart Failure and Associated Comorbidities on HDL

**Figure 1:**
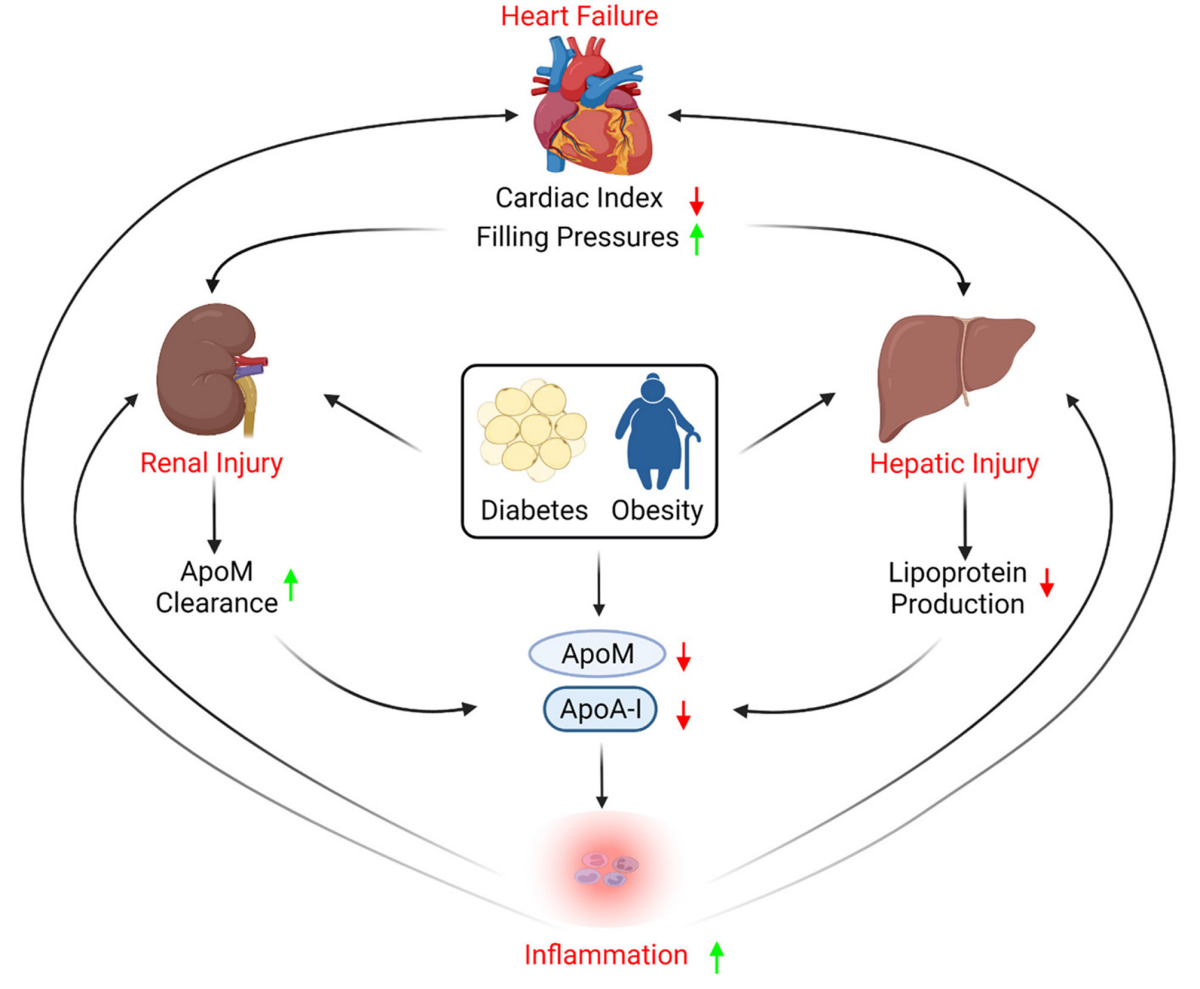
Apolipoproteins role in heart failure progression. Heart failure causes reduced cardiac index and increased filling pressures, which subsequently leads to hepatic injury that can affect apolipoprotein production. In addition, heart failure-induced kidney injury may increase renal excretion of apolipoprotein M (ApoM). Co-morbidities such as diabetes and obesity are also known to reduce circulating apolipoproteins, contributing to inflammation, thus exacerbating kidney and hepatic injury, and provoking further cardiac dysfunction.

Advanced HF is a multisystem syndrome often identified in patients with multiple cardiometabolic comorbidities; hence, both HF and its associated comorbidities can have complex effects on lipoprotein biology. Hepatic, renal, and gastrointestinal malperfusion secondary to reduced cardiac index and increased filling pressures all contribute to a vicious cycle of decreased nutritional intake, increased inflammation, metabolic stress, perturbations that can have important effects on lipoprotein homeostasis (Figure 1).

### Effects of Chronic Inflammation on HDL

HF is characterized by a chronic inflammatory state. While the increase in pro-inflammatory cytokines in HF has been well-documented, there is still debate regarding the extent to which increased cytokines are directly responsible for deleterious results or are simply a reflection of the ongoing pathophysiological processes (46–49). Nevertheless, chronic inflammatory states, such as that observed in HF, can affect plasma HDL levels, composition, and overall function. For instance, plasma HDL contains lower cholesterol ester levels, higher free cholesterol, triglycerides, and fatty acids under inflammatory states (50). Moreover, inflammation strips HDL from key proteins that are important for its normal function (e.g., lecithin-cholesterol acyltransferase (LCAT), cholesteryl ester transfer protein, and transferrin), as well as certain important apolipoproteins (e.g., apolipoproteins A-I and M) (51–55). Apolipoprotein A-I (ApoA-I) is the primary mediator of cholesterol efflux, the key rate-limiting step of reverse cholesterol transport, and the main protein component of HDL particles (56–59). In the same context, apolipoprotein M (ApoM), a cardioprotective apolipoprotein (45), is a negative acute response protein, levels of which decrease in response to inflammation and infection (52, 60, 61). The decrease in HDL levels and alteration of its structural composition in inflammatory states impair the reverse cholesterol transport process and HDL's anti-inflammatory and anti-oxidant properties. In the long run, this can lead to the development of atherosclerosis and increased risk of CVD and HF.

How inflammation affects HDL particle number and composition is not very well-understood. In mice, endotoxin directly impairs active cholesterol efflux by ATP-binding cassettes A1 and G1 (ABCA1 and ABCG1) transporters, as well as scavenger receptor class B type I (SR-B1) mediated passive diffusion (62–64). Meanwhile, inflammatory cytokines, such as tumor necrosis factor alpha (TNF-α) and interleukins 1 and 6 (IL-1β, IL-6), upregulate the expression of endothelial lipase (EL), which exhibits an inverse association with HDL levels (65, 66). Badellino et al. showed that experimental administration of low-dose endotoxin in humans decreases HDL phospholipid, corresponding with EL peak concentration (67). Tietge et al. (68) reported that mice that overexpress secretory phospholipase A2 have changes in HDL composition, and under inflammatory conditions, exhibit increased HDL catabolism. Interestingly, in a murine model of pressure overload-induced HF, EL knockout exacerbated cardiac dysfunction compared to wild-type controls, consistent with the hypothesis that EL provides an alternative pathway for free fatty acid uptake as a source of energy and protects the failing myocardium (69). Thus, it is plausible that chronic inflammation may be upregulating the expression of EL, which leads to HDL catabolism to release fatty acids to the energy-starved myocardium at the expense of other cardioprotective components of the HDL particle.

### Effects of Renal Dysfunction on HDL

Normal renal function is crucial for proper HDL function (70). Renal dysfunction induces pathologic alterations in lipoprotein metabolism in general, and HDL in particular (71). HF can induce renal dysfunction, which is a strong independent predictor of poor cardiovascular outcomes (72, 73). Cardiorenal syndrome is a term that describes the mutual interaction between the heart and kidneys, considering that injury to one of the organs usually causes dysfunction of the other (74).

Renal dysfunction and the associated chronic inflammatory state present in cardiorenal syndrome correlate with increased oxidative stress across multiple systems (75). Oxidized HDL (ox-HDL) is a modified HDL observed during conditions of increased oxidative stress and reduced anti-oxidant capacity present in cardiorenal syndrome (76, 77). Various HDL and ApoA-1 post-translational modifications can result in ox-HDL formation (76, 78, 79), which has been linked to an increased risk of cardiovascular events (80). Myeloperoxidase (MPO) can modify ApoA-I leading to ox-HDL that is less avid in its ability to bind SR-BI receptors and dysfunctional for normal cholesterol efflux activity (81, 82). Conversely, hypochlorite-generated ox-HDL exhibits increased affinity toward SR-BI, albeit with less cholesterol efflux capacity than normal HDL (83). We propose that various post-translational modifications (for example, MPO adducts) might alter specific ox-HDL characteristics (for example, higher vs. lower affinity toward SR-BI); nonetheless, renal dysfunction can contribute toward “dysfunctional” HDL particles. Moreover, ox-HDL exhibits diminished endothelial nitric oxide synthase (eNOS) mediated endothelial protective function as well as anti-apoptotic activity, which leads to impaired endothelial repair and increased pro-inflammatory activation (84–86).

In a proteomic analysis of HDL in uremic patients, isolated HDL particles lost their anti-inflammatory properties and induced the production of inflammatory cytokines (87). HDL isolated from these patients contained high levels of serum amyloid A (SAA), a protein known to promote inflammatory cytokine production and impair the anti-inflammatory capacity of HDL (87). Furthermore, HDL from uremic patients may contribute to the systemic inflammatory state in chronic kidney disease patients by decreasing apoptosis of polymorphonuclear leukocytes (88).

### Effects of Diabetes on HDL

Diabetes is a pathophysiological process that can significantly impact the biogenesis of HDL, and cause alterations in myocardial metabolism, impairing metabolic flexibility and leading to diabetic cardiomyopathy (89). Oxidative stress, intramyocardial inflammation, cardiac fibrosis, and cardiac apoptosis all contribute to diabetic cardiomyopathy (90), which can in theory be mitigated by the anti-inflammatory and anti-oxidative functions of HDL.

In diabetes, hyperglycemia-induced advanced glycation end products, oxidative stress, and inflammation can negatively affect normal HDL function and composition, potentially contributing to an increased risk of HF (91). Glycated HDL loses atheroprotective properties and cholesterol-accepting capacity, leading to the acceleration of atherosclerosis (92). HDL isolated from diabetic patients is also rendered ineffective concerning endothelial protective function (93). Many of the pleiotropic effects of HDL are attributed to ApoM-bound sphingosine-1-phosphate (S1P), which is diminished in diabetic patients mainly due to glycation of ApoM that results in the impaired binding capacity to S1P (94).

### Effect of Obesity on HDL

Obesity has been established as a major risk factor for hypertension, CVD, and left ventricular hypertrophy, all risk factors for the development of HF (95). Obesity is associated with reduced HDL-C. In a large cross-sectional study, HDL-C is inversely associated with body mass index (BMI) (96). Obesity can also affect HDL subclasses and metabolism likely reflecting an underlying change in key HDL proteins and lipids (97, 98). Plasma ApoA-I exhibits a linear inverse correlation with BMI (99), while ApoM is also reduced in obese individuals (100) and is inversely associated with non-alcoholic fatty liver disease (NAFLD) (101), another comorbidity associated with obesity and an emerging risk factor for HF, in particular HFpEF (102).

Proteomic studies of HDL in patients with obesity and other comorbidities have also been informative. In NAFLD patients, quantitative changes occur in the HDL proteome, relative to morbidly obese patients without steatosis (103). One challenging aspect of studying comorbidities related to obesity is selecting the best control or reference population. Comparing obese patients with comorbidities of obesity vs. more metabolically healthy obese patients will likely minimize differences between groups. Another challenge is that many unmeasured confounders might be associated with obesity. Further prospective studies are required to unravel the complex interactions between obesity and its comorbidities, including HF, and how these interactions might be mediated by lipoproteins. In particular, the need for prospective studies is highlighted by the focus of older literature on HDL subclasses, and new studies suggesting that meals of various fat compositions can acutely affect the HDL proteome (104). Altogether, obesity itself, or its comorbidities, may alter HDL proteomic and lipidomic contents, impairing potential cardioprotective functions. This area is both complex and rapidly evolving.

### Effect of Atrial Fibrillation on HDL

Metabolic disease, obesity, and HF can also result in arrhythmias. The most common arrhythmia observed in patients is atrial fibrillation (AF). Low baseline HDL-C is associated with an increased risk of AF (105–108). AF is associated with reduced HDL quality as AF was associated with reduced HDL cholesterol efflux capacity, HDL-particle number, ApoA-I levels, and reduced LCAT activity; interestingly, all these indices improved following the restoration of sinus rhythm (109). Further validation of these findings would be encouraging, especially because the mechanistic link between AF and HDL remains unclear, particularly in the acute setting. One possible theory is the role HDL plays in myocardial membrane stabilization (110). In the more chronic setting, other HDL attributes including anti-inflammatory, anti-oxidant, and anti-atherogenic properties could interact with AF development and severity.

### Effect of Aging on HDL

Aging is a well-established risk factor for the development and progression of HF, resulting from the deterioration of both cardiac structure and function, as well as the high risk of co-morbidities. Elderly patients have increased HDL oxidation, which can impair the normal protective capacity against LDL oxidation, and lead to the acceleration of atherosclerosis and CVD, both risk factors for HF (111).

Holzer et al. (112) compared HDL isolated from healthy young and elderly patients and found that aging alters HDL composition and function. HDL from elderly subjects had higher SAA and sphingomyelin, while levels of total cholesterol were reduced (112). Furthermore, HDL isolated from older patients demonstrated reduced cholesterol efflux capacity, principally through the ABCA1 pathway (113). In the same context, aged murine models have exhibited reduced ApoM secretion from the liver, with consequent impairment of S1P signaling, which reduces resistance to injury-induced vascular leak and precipitates organ fibrosis (114).

Oxidative stress is one of the main pathophysiological processes associated with aging (115) and is involved in the development of HF (116). PON1 is one of the most prominent antioxidant components of HDL (112, 117, 118). In elderly patients, it has been shown that PON1 activity and ApoE levels, both having important antioxidant properties, are diminished (112, 119). Overall, these studies suggest that aging may alter HDL structure and properties, resulting in reduced antioxidant capacity and cholesterol efflux, which can contribute to higher susceptibility to CVD and advance processes associated with HF mortality.

Inflammation, renal dysfunction, diabetes, obesity, atrial fibrillation, and aging can either occur antecedent to HF or comorbid with it. These HF comorbidities, and others, can have a tremendous impact on lipid metabolism and HDL biology, which in turn may impact disease progression. In the next section, we discuss how changes in HDL may alter the development of HF or HF outcomes.

## Salutary Effects of HDL on Prevention and Outcomes in Heart Failure

### Atheroprotective Functions of HDL

Atherosclerotic CVD can lead to ischemic cardiomyopathy, which is a major clinical cause of HF (120). LDL-C is a critical, causal factor in the pathogenesis of CVD (121). In animal models, HDL has been shown to have a protective role against the development of atherosclerosis and CVD (122). HDL exerts its protective effect on vascular endothelium mainly through stimulation of eNOS increasing NO production (123, 124). HDL is critical for the reverse cholesterol transport process, which removes excess cholesterol from the atherosclerotic plaques, reducing the risk and progression of CVD (125, 126). Additionally, HDL has anti-apoptotic, anti-inflammatory, and antithrombotic protective properties on the vascular endothelium (34, 124, 127).

### Cardioprotective Functions of HDL

Both *in vitro* and *in vivo* models have repeatedly demonstrated multiple cardioprotective properties of HDL particles on many levels. HDL has shown a direct protective effect on cardiomyocytes and endothelial cells, independent of its effect on the coronary vasculature or atherosclerosis (16, 26, 128–131). It has been proposed that HDL mediates direct action on cardiomyocytes through its different array of apolipoproteins (e.g., ApoA-I and ApoM), which interact with different receptors expressed on cardiomyocytes regulating various intracellular signaling pathways. Furthermore, HDL could also indirectly protect cardiomyocytes through its systemic and local anti-inflammatory and anti-oxidative effects (132, 133).

In murine models, HDL inhibited mechanical stress-induced myocardial cell hypertrophy and autophagy through downregulation of the angiotensin II type 1 receptor (129). Angiotensin II receptors are upregulated on cardiomyocytes exposed to mechanical stress, and blockade of the renin-angiotensin pathway is a sine qua non of HF therapy. Downregulation of these receptors by HDL would be an important mechanism by which this particle may improve HF outcomes. Further, multiple *in vitro* studies show that HDL also protected against doxorubicin-induced cell injury on cultured cardiomyocytes (26, 134, 135), mostly through reducing doxorubicin-induced apoptosis. These studies are clinically important given that anthracyclines, such as doxorubicin, remain an important cause of cardiotoxicity and clinical HF.

HDL has also been associated with the preservation of endothelial barrier integrity. HDL increases NO production in endothelial cells, which enhances endothelial vasodilation and preserves endothelial integrity mainly through an SR-BI-dependent mechanism (136–140). It also modulates the contractile state of subjacent myocytes *via* paracrine mechanisms (141). Additionally, HDL contributes to endothelial repair by increasing the number and function of endothelial progenitor cells at sites of endothelial injury (128). Moreover, HDL-carried glycosphingolipids have demonstrated an anti-apoptotic capacity against stress-induced endothelial death (142). Van Linthout et al. (130) reported that HDL protects against myocardial dysfunction and hyperglycemia-induced cardiomyocyte apoptosis in diabetic murine models mainly *via* the phosphoinositide 3-kinase / protein kinase B (PI3K/Akt) pathway. Altogether, these studies suggest multiple mechanisms by which HDL may directly protect cardiomyocytes in the failing myocardium.

### HDL Anti-inflammatory Properties

It has previously been established that systemic inflammatory mediators (e.g., C reactive protein (CRP), TNF-α) can contribute to the development of HF, and inflammation can induce cardiomyocyte apoptosis and endothelial dysfunction (143). Multiple studies have demonstrated anti-inflammatory properties of HDL. For instance, HDL inhibits endothelial activation and decreases the expression of adhesion molecules (e.g., VCAM-1 and ICAM-1), which prevents the recruitment of leukocytes in response to myocardial cell injury, and can attenuate the insult due to reduced chemokine secretion and impede further recruitment of inflammatory cells. HDL also blocks T-cell binding and activation of monocytes, which results in diminished production of pro-inflammatory cytokines (144–146).

Moreover, recent studies suggest that the NOD-, LRR- and pyrin domain-containing protein 3 (NLRP3) inflammasome plays a role in the development of atherosclerosis, and it has also been tied to post-ischemic remodeling, and HF (147–149). HDL can suppress the activation of the NLRP3 inflammasome, likely by simultaneous downregulation of IL-1β, and reduced activation of caspase 1 (150). Other anti-inflammatory properties of HDL are promoting the expression of anti-inflammatory cytokines, such as transforming growth factor-β2 (TGF-β2) in endothelial cells (151, 152) and neutralizing pro-inflammatory activity of both, IL-6 and CRP (153). In summary, these and other anti-inflammatory properties of HDL merit further exploration and offer a variety of targets for developing pharmacologic therapies.

### HDL Anti-oxidative Properties

One of the hallmarks of HF pathophysiology is stress-induced myocardial cell death with subsequent proliferation, fibrosis, and remodeling (154, 155). HDL has demonstrated many antioxidant properties that may combat these processes. Treating cultured cardiomyocytes with HDL protects against stress-induced cell death (156). This effect has been suggested to be mediated by the anti-oxidative enzyme PON1 (157–160). Another antioxidant enzyme present on HDL is the platelet-activating factor acetylhydrolase that induces the hydrolysis of fatty acids and phospholipids peroxides (161, 162). Furthermore, HDL blocks eNOS uncoupling in myocardial cells, reducing the formation of reactive oxygen species (163–166). In addition, HDL-associated lipoproteins (ApoA-I, ApoA-II, ApoA-IV, ApoE, etc.) neutralize the remaining phospholipid hydroperoxides transferred to HDL (167). Finally, HDL can also indirectly reduce oxidative stress secondary to its anti-inflammatory properties discussed previously (168).

### HDL Anti-fibrotic Properties

HDL can protect against myocardial fibrosis through inhibition of the pro-fibrotic transforming growth factor-β1 (TGF-β1), which induces collagen production and deposition in the myocardium of murine models (169, 170). In a study on aortic endothelial cells *in vitro*, HDL reduced TGF-β1-induced endothelial-mesenchymal transition and attenuated fibrosis of the vascular wall in response to various insults (171). Alternatively, HDL may exhibit anti-fibrotic properties by binding and potentially sequestering S1P (through ApoM). Although this mechanism has not been directly demonstrated in the myocardium, this type of biology has been demonstrated in the retina, where ApoM can act as a negative regulator of S1P (172).

### Cardioprotective Role of ApoA-I/SR-BI Axis

ApoA-I, the most abundant protein constituent of HDL, is involved in the systemic anti-inflammatory and anti-oxidative cardioprotective properties of HDL (173). ApoA-I is the main ligand of SR-BI and thus is very important for the cardioprotective functions of HDL previously described. Low ApoA-I levels are associated with left ventricular dysfunction and adverse outcomes in patients with non-ischemic HF (173, 174). Gombos et al. have shown that ApoA-I is inversely associated with NT-proBNP and mortality in HF (175). Similarly, Florvall et al. (176) have suggested that serum ApoA-I can predict CVD and mortality in elderly men.

ApoA-I's cardioprotective properties may be related to its anti-inflammatory and antioxidative properties. ApoA-I attenuates inflammation and is inversely correlated with CRP and fibrinogen levels (173). In addition, it blocks neutrophil activation and expression of the surface adhesion proteins that regulate leukocyte migration (177, 178). Bursill et al. (144) showed that when mice were injected with ApoA-I, the expression of chemokine receptors involved in leukocyte migration was significantly reduced. ApoA-I can also enhance the proliferation of endothelial progenitor cells and stimulate angiogenesis through the cell surface F1-ATP synthase, a high-affinity receptor of ApoA-I (179). Moreover, ApoA-I accelerates endothelial regeneration and prevents transplant vasculopathy in murine models (132, 180).

**Figure 2:**
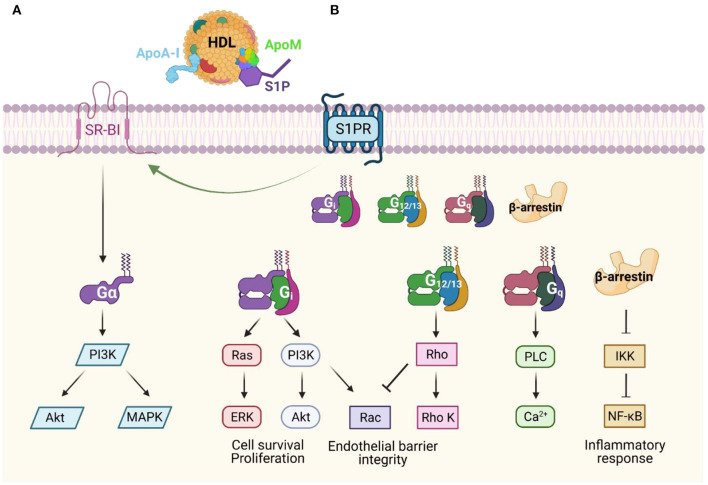
Apolipoprotein-dependent signal transduction pathways **(A)** Apolipoprotein A-I (ApoA-I) is the major component of high-density lipoproteins (HDL), and it binds scavenger receptor class B type I (SR-BI), which mediates selective uptake of cholesterol. SR-BI may also stabilize HDL particles allowing access to S1P receptors or activate other signaling cascades. **(B)** HDL and apolipoprotein M (ApoM)-dependent activation of S1P receptors (S1PR) leads to downstream G-protein coupled receptor signaling in both endothelial cells and cardiomyocytes. This signaling promotes diverse physiological responses including maintenance of endothelial barrier integrity, promotion of cell survival, and anti-inflammatory effects. Modified from (240–242).

ApoA-I binds to SR-BI, which is mainly expressed in the liver. SR-BI mediates selective uptake of cholesterol, as well as HDL lipid hydroperoxides, and plays a major role in modulating HDL composition and therefore its function (Figure 2A). Muthuramu et al. described a cardioprotective role of SR-BI (181). They performed a study using SR-BI knockout mice that received either adeno-associated virus 8 (AAV8) expressing SR-BI (via a hepatocyte-specific promoter) or a control AAV8. Notably, when SR-BI knockout mice are exposed to pressure overload, they develop worse pathological ventricular hypertrophy, interstitial and perivascular fibrosis, and myocardial apoptosis than control mice. Interestingly, in mice that received AAV8-SR-BI, the plasma lipoprotein profile normalizes, attenuates cardiac dysfunction, and mortality is lower compared to mice that received Null injection. In addition, mice that underwent SR-BI gene transfer had lower oxidative stress than those that did not (181). Similarly, Durham et al. demonstrated that pretreatment with HDL protects against myocardial cell necrosis *via* the PI3K/Akt pathway (131). This finding was not observed in SR-BI knockout cells, suggesting that SR-BI is the upstream mediator of the PI3K/Akt signaling in cardiomyocytes and that HDL could be mediating this effect through interaction with SR-BI *via* ApoA-I (131). These studies suggest that ApoA-I, *via* SR-BI, may be an important mediator of the cardio-hepatic axis.

### Cardioprotective Role of the ApoM/S1P/S1PR Axis

ApoM is an apolipoprotein that binds S1P *via* its hydrophobic binding pocket, is secreted mostly by hepatocytes, and to a lesser extent by renal proximal tubular cells (182, 183). Although ApoM is only found in 5% of HDL particles, it exerts many of the beneficial effects of HDL through S1P signaling (Figure 2B). ApoM acts as a chaperone for S1P carrying about 70% of plasma S1P in the circulation, as well as increasing its efflux from erythrocytes to HDL (184). ApoM that is secreted from the proximal tubular cells in the kidney also prevents renal excretion of S1P (185).

Recent studies indicate a potential protective role of ApoM on atherosclerosis and CVD (186–188). ApoM has a protective effect on vascular endothelium as ApoM transgenic LDL receptor knock out mice developed smaller atherosclerotic lesions than control mice (189, 190). Atheroprotective functions of ApoM on vascular endothelium are likely mediated by S1P through S1P receptor 1 (S1PR1) signaling (191). ApoM also has a significant anti-inflammatory effect *in vivo* and *in vitro* mainly mediated by the S1P/S1PR axis (192).

Multiple mechanisms have been invoked for how ApoM may improve myocardial health or delay disease progression. Recent studies in murine models have demonstrated that ApoM/S1P enhances endothelial barrier function and improves cardiac outcomes through different signaling pathways. For example, in LPS-treated mice, ApoM attenuated LPS-induced organ injury as well as cardiomyocyte cell death *via* PI3K/Akt downstream of S1PR1/3 (193). Moreover, *in vitro* studies of human umbilical vein endothelial cells showed that ApoM/S1P markedly reduced pro-inflammatory cytokines, including TNF-α, inhibiting the inflammatory response, and reduced endothelial injury in a PI3K/Akt and S1PR2 dependent manner (194). Furthermore, ApoM knockout mice demonstrate impaired endothelial barrier integrity compared to wild-type mice (195). Reconstitution of plasma ApoM/S1P or treatment with an S1PR1 agonist rapidly reversed the vascular leak and restores endothelial integrity (195). S1P, acting through the receptors 1-3 (S1PR1-3), plays a crucial role in the regulation of the endothelial cell cytoskeleton, and is necessary for its proper function as well as new vessel formation. Multiple studies have demonstrated S1P to be a significant mediator of angiogenesis due to its potent chemoattractant properties for endothelial cells (196–198). S1P was found to have a higher capacity for stimulation of endothelial cell migration than known molecules such as vascular endothelial growth factor or basic fibroblast growth factor (199). Furthermore, S1P acting mainly *via* S1PR1 and S1PR3 have been repeatedly demonstrated to be crucial for endothelial migration (200, 201), endothelial integrity (202–204), and normal barrier function (205, 206). S1P effects on endothelial cells are mainly mediated by pathways involving Rho GTPases (207–209) as well as the PI3K/Akt pathway (210).

In addition to its protective role on the endothelium, S1P has shown multiple cardioprotective properties. Zhang et al. (211) showed that S1P signaling through S1PR1 in murine models activated the downstream PI3K/Akt pathway and attenuated myocardial cell injury in response to severe hypoxic stress. Similarly, Means et al. found that stimulation of S1PR2 and S1PR3 receptor activates PI3K/Akt pathway and protects against ischemia-reperfusion injury (212). Theilmeier et al. have also demonstrated that HDL and S1P, both acting through S1PR3, and NO-dependent mechanisms, protect against ischemia reperfusion-induced myocardial injury in *ex vivo* and *in vivo* mouse models (25). They also found that S1P reduced neutrophil recruitment to the site of injury and decreased cardiomyocyte apoptosis (25). Furthermore, S1PR2 showed some cardioprotective properties as well by activating signal transducer and activator of transcription 3 (STAT3) through ERK1/2 and Src-dependent mechanisms (26). STAT3 is important for myocardial adaptation to stress and has been shown to preserve cardiac function through its anti-apoptotic and anti-fibrotic effects (213–218). While these data support multiple mechanisms for S1P-mediated anti-apoptotic effects on cardiomyocytes through multiple S1P receptors, both in our experience and others, it is challenging to detect any significant S1PR2 mRNA expression in the myocardium in mice (219).

These studies support the concept that ApoM, *via* S1P, can reduce vascular leak, inflammation, and promote cell survival, all of which are likely critical targets for multiple organs in the syndrome of HF. Recently, our group measured circulating ApoM across 3 major HF cohorts comprising nearly 2,500 patients. In our study, reduced ApoM levels were significantly associated with the risk of all-cause mortality (45). These associations were independent of HDL-C and ApoA-I, natriuretic peptide levels, etiology of HF, and a commonly used HF risk score, and were observed in both HFrEF and HFpEF. Although we demonstrated a strong correlation between ApoM and S1P on HDL particles, mediation analysis suggested that ApoM could also have effects independent of S1P. Pathway analysis demonstrated that ApoM showed that the acute phase response was not only the most significant pathway associated with ApoM but also that ApoM was inversely associated with inflammation, as predicted by murine studies. In a follow-up study, we screened the plasma proteome to identify proteins that mediated the effect of diabetes on HFpEF outcomes. The only protein that fulfilled the criteria of this a priori analysis was ApoM, which was shown to mediate an astounding 70% of the effect of diabetes on HFpEF outcomes (220).

## Novel HDL Therapeutics in Cardiovascular Diseases

Multiple studies have shown a promising role for HDL-targeted therapies in HFrEF (221), HFpEF (221, 222), and diabetic cardiomyopathy murine models (223). In these animal models, HDL reversed pathologic features of myocardial hypertrophy, fibrosis, and stimulating reverse remodeling in pre-established HF. Multiple synthetic compounds have been designed to mimic the bioactive molecules of HDL and replicate their cardioprotective functions.

ApoA-I Milano is an ApoA-I mutant first described in Northern Italy in 1980 (224, 225). Heterozygous carriers of the mutation were thought to exhibit increased life expectancy and believed to develop atherosclerosis at lower rates compared to the normal population (226, 227). MDCO-216 is a recombinant HDL formulation of ApoA-I Milano in combination with phospholipids, which has been used to study ApoA-I Milano's potential therapeutic effects (183, 228–230).

Mishra et al. (222) reported that MDCO-216 attenuated cardiac hypertrophy, increased capillary density, and decreased interstitial fibrosis in murine models. In a subsequent study, Mishra et al. showed similar results of MDCO-216 in murine models of hypertension-associated cardiac hypertrophy (170). Aboumsallem et al. have demonstrated that MDCO-216 improves systolic and diastolic dysfunction, reduces myocardial fibrosis, and enhances myocardial vascularity in mice with HF (221). Further, Aboumsallem et al. showed that mice with diabetic cardiomyopathy that were treated with MDCO-216 presented regression of myocardial dysfunction and pathological cardiac remodeling (223). Altogether, these studies suggest MDCO-216 might be useful for HFrEF, HFpEF, or diabetic cardiomyopathy.

ApoA-I gene therapy strategies have also been employed in HF rodent models. Gordts et al. (231) evaluated if selective gene transfer may protect against the development of HF. In LDL receptor-deficient subjects to experimental MI, viral-mediated gene transfer of ApoA-I resulted in reduced infarct expansion and inhibition of left ventricular dilatation compared with controls. Similarly, Amin et al. studied the effect of selective AAV8-human ApoA-I (AAV8-ApoA-I) gene transfer on cardiac remodeling, induced by transverse aortic constriction in LDL deficient mice (232). They reported that AAV8-ApoA-I transduced mice had significantly attenuated septal wall thickness, cardiomyocyte cross-sectional area, and interstitial cardiac fibrosis compared to control mice, indicating reduced remodeling, and preserved systolic function reserve. Diastolic function was also significantly improved in mice transduced with the ApoA-I AAV8 (232).

ApoA-I mimetic peptides have also shown promise in preventing or attenuating myocardial dysfunction in murine models of MI and sepsis. Hamid et al. (233) have demonstrated that the ApoA-I mimetic peptide L-4F prevents prolonged and excessive inflammation after MI and improves post-MI LV remodeling. L-4F suppressed proliferation of myocardial pro-inflammatory monocytes and macrophages in murine models of reperfused MI (233). They suggested that L-4F could be used as a therapeutic adjunct in humans with MI to limit inflammation and alleviate the progression to HF (233). Another ApoA-I mimetic peptide D-4F has been also shown to improve vascular function, decrease myocardial inflammation, and restore angiogenic systemic sclerosis in murine models (234). Moreover, the ability of ApoA-I mimetic peptides to reduce sepsis-induced myocardial injury was studied by Moreira et al. (235). They demonstrated that the novel ApoA-I mimetic peptide D-4F reduced inflammation, attenuated vascular permeability, preserved myocardial function, and baroreceptor sensitivity in murine models of sepsis.

The AEGIS-I trial (Apo-AI Event Reducing in Ischemic Syndromes I), a multi-center, randomized, double-blind, placebo-controlled 2b trial assessed the safety of CSL112, an infusible plasma-derived ApoA-I, in patients with myocardial infarction (236). CSL112 was generally safe and well-tolerated (236). Currently, the AEGIS-II trial is underway to evaluate whether CSL112 can reduce the risk of major adverse cardiovascular events (237). To our knowledge, there are no active trials of CSL112 in human HF, although the hypothesis that CSL112 may benefit patients with acute HF should be pursued in randomized-controlled clinical trials (238).

Beyond therapeutics targeting only ApoA-I, Swendeman et al. (239) developed a recombinant ApoM fused to the constant domain of immunoglobins (ApoM-Fc) to prevent its rapid degradation. When this novel protein was tested in multiple systems, ApoM-Fc selectively activated S1PR1, leading to enhanced endothelial barrier integrity and downstream eNOS-dependent secretion of NO and subsequent vasodilation, which could be used therapeutically to control hypertension. In addition, they demonstrated improved outcomes in murine models of myocardial ischemia/reperfusion and stroke, by promoting endothelial function and reducing further tissue inflammation.

Altogether, apolipoprotein A-I and ApoM based therapeutics have demonstrated potential in preclinical models of cardiac dysfunction. Unfortunately, to our knowledge, the human translation of these therapeutics has not been tested in randomized controlled clinical trials in HF. In addition, understanding regarding the synergistic potential of these apolipoproteins (A-I and M), as well as other functions of HDL, remains poorly understood.

## Conclusions

HDL apolipoproteins remain a promising therapeutic target in patients with HF. The advances in proteomic and lipidomic technologies have permitted the discovery of HDL components, and assessment of their impact on HF pathophysiology, predominantly in preclinical models. ApoA-I and ApoM are especially promising as they have shown multiple cardioprotective properties in murine models. Further studies are needed to elucidate the functional properties of HDL proteomic and lipidomic components and to explore possible therapeutic targets in patients with HF.

## Author Contributions

All authors listed have made a substantial, direct, and intellectual contribution to the work and approved it for publication.

## Funding

AJ was supported by R01 HL155344 and K08HL138262 from the NHLBI and by the Diabetes Research Center at Washington University in St. Louis of the National Institutes of Health under award number P30DK020579, as well as the NIH grant P30DK056341 (Nutrition Obesity Research Center), and by the Children's Discovery Institute of Washington University (MC-FR-2020-919) and St. Louis Children's Hospital. ZG was supported by the American Heart Association Postdoctoral Fellowship (898679).

## Conflict of Interest

AJ has a patent for fusion protein nanodiscs and lipase inhibitors for the treatment of heart failure and has received grant support from AstraZeneca. The remaining authors declare that the research was conducted in the absence of any commercial or financial relationships that could be construed as a potential conflict of interest.

## Publisher's Note

All claims expressed in this article are solely those of the authors and do not necessarily represent those of their affiliated organizations, or those of the publisher, the editors and the reviewers. Any product that may be evaluated in this article, or claim that may be made by its manufacturer, is not guaranteed or endorsed by the publisher.
